# A rasch model to test the cross-cultural validity in the positive and negative syndrome scale (PANSS) across six geo-cultural groups

**DOI:** 10.1186/2050-7283-1-5

**Published:** 2013-03-11

**Authors:** Anzalee Khan, Christian Yavorsky, Stacy Liechti, Mark Opler, Brian Rothman, Guillermo DiClemente, Luka Lucic, Sofija Jovic, Toshiya Inada, Lawrence Yang

**Affiliations:** 1 ProPhase, LLC, New York, NY United States of America; 2 CROnos Clinical Consulting Services, Hamilton, NJ United States of America; 3 The PANSS Research Institute, Inc, New York, NY United States of America; 4 Nathan S. Kline Institute for Psychiatric Research, Orangeburg, NY United States of America; 5 Manahttan Psychiatric Center, Wards Island, NY United States of America; 6 New York University, School of Medicine, New York, NY United States of America; 7 Pratt Institute, Brooklyn, NY United States of America; 8 Department of Epidemiology, Columbia University, New York, NY United States of America; 9 Institute of Neuropsychiatry, Seiwa Hospital, Shinjuku-ku, Tokyo, Japan

## Abstract

**Background:**

The objective of this study was to examine the cross-cultural differences of the PANSS across six geo-cultural regions. The specific aims are (1) to examine measurement properties of the PANSS; and (2) to examine how each of the 30 items function across geo-cultural regions.

**Methods:**

Data was obtained for 1,169 raters from 6 different regions: Eastern Asia (n = 202), India (n = 185), Northern Europe (n = 126), Russia & Ukraine (n = 197), Southern Europe (n = 162), United States (n = 297). A principle components analysis assessed unidimensionality of the subscales. Rasch rating scale analysis examined cross-cultural differences among each item of the PANSS.

**Results:**

Lower item values reflects items in which raters often showed less variation in the scores; higher item values reflects items with more variation in the scores. Positive Subscale: Most regions found item P5 (Excitement) to be the most difficult item to score. Items varied in severity from −0.93 [item P6. Suspiciousness/persecution (USA) to 0.69 item P4. Excitement (Eastern Asia)]. Item P3 (Hallucinatory Behavior) was the easiest item to score for all geographical regions. Negative Subscale: The most difficult item to score for all regions is N7 (Stereotyped Thinking) with India showing the most difficulty Δ = 0.69, and Northern Europe and the United States showing the least difficulty Δ = 0.21, each. The second most difficult item for raters to score was N1 (Blunted Affect) for most countries including Southern Europe (Δ = 0.30), Eastern Asia (Δ = 0.28), Russia & Ukraine (Δ = 0.22) and India (Δ = 0.10). General Psychopathology: The most difficult item for raters to score for all regions is G4 (Tension) with difficulty levels ranging from Δ = 1.38 (India) to Δ = 0.72.

**Conclusions:**

There were significant differences in response to a number of items on the PANSS, possibly caused by a lack of equivalence between the original and translated versions, cultural differences among interpretation of items or scoring parameters. Knowing which items are problematic for various cultures can help guide PANSS training and make training specialized for specific geographical regions.

## Background

Psychopathology encompasses different types of conditions, causes and consequences, including cultural, physical, psychological, interpersonal and temporal dimensions. Diagnosing and measuring the severity of psychopathology in evidence-based medicine usually implies a judgment by a clinician (or, rater) of the experience of the individual, and is generally based on the rater’s subjective perceptions [1]. Structured or semi-structured interview guides have aided in increasing rater consistency by standardizing the framework in which diagnostic severity is measured. In clinical trials, good inter-rater reliability is central to reducing error variance and achieving adequate statistical power for a study – or at least preserving the estimated sample size outlined in the original protocol. Inter-rater reliability typically is established in these studies through rater training programs to ensure competent use of selected measures.

The Standards for Educational and Psychological Testing (American Educational Research Association, AERA [2]) indicate that test equivalence include assessing construct, functional, translational, cultural and metric categories. Although, many assessments used in psychopathology have examined construct, functional, translational and metric categories of rating scales, except for a handful of studies [3, 4], the significance of clinical rater differences across cultures in schizophrenia rating scales has rarely been investigated. There is ample research demonstrating the penchant for clinical misdiagnosis and broad interpretation of symptoms between races, ethnicities, and cultures, usually Caucasian American or European vis-à-vis an “other.” For example, van Os and Kapur [5], and Myers [6] point to a variation in cross-cultural psychopathology ratings. The presence of these findings suggests that the results of psychiatric rating scales may not adequately assess cultural disparities not only in symptom expression but also in rater judgment of those symptoms and their severity. Several primary methods have been championed in the past decade as means to aid in the implementation of evaluation methods in the face of cultural diversity [7–9]. These approaches, still in their infancy, have yielded positive results in the areas of diagnosis, treatment, and care of patients, but they still require reevaluation and additional adjustment [10–12]. As clinical trials become increasingly global, it is imperative to understand the limitations of current tools and to adapt, or to augment methods where, and when necessary.

One of the most widely used measures of psychopathology of schizophrenia in clinical research is the Positive and Negative Syndrome Scale (PANSS) [13–15]. Since its development, the PANSS has become a benchmark when screening and assessing change, in both clinical and research patients. The strengths of the PANSS include its structured interview, robust factor dimensions, reliability [13, 16, 17], availability of detailed anchor points, and validity. However, a number of psychometric issues have been raised concerning assessment of schizophrenia across languages and culture [18]. Given the widespread use of the PANSS in schizophrenia and related disorders as well as the increasing globalization of clinical trials, understanding of the psychometric properties of the scale across cultures is of considerable interest.

Most international prevalence data for mental health is difficult to compare because of diverse diagnostic criteria, differences in perceptions of symptoms, clinical terminology, and the rating scales used. For example, in cross-cultural studies with social variables, such as behavior, it is often assumed that differences in scores can be compared at face value. In non-psychotic psychiatric illnesses, cultural background has been shown to have substantial influence on the interpretation of behavior as either normal or pathological [19]. This suggests that studies using behavioral rating scales for any disorder should not be undertaken in the absence of prior knowledge about cross-cultural differences when interpreting the behaviors of interest.

There are a number of methodological issues when evaluating cross-cultural differences using results obtained from rating scales [20–23]. Rasch models have been used to examine and account for, cross-cultural bias [24]. Riordan and Vandenberg [25] (p. 644) discussed two focal issues in measurement equivalence across cultures, (1) whether rating scales elicit the same frame of reference in culturally diverse groups, and (2) whether raters calibrate the anchor points (or scoring options) in the same manner. Having non-equivalence in rating scales among cultures can be a serious threat to the validity of quantitative cross-cultural comparison studies as it is difficult to tell whether the differences observed are reflecting reality. To guide decision-making on the most appropriate differences within a sample, studies advocate more comprehensive analyses using psychometric methods such as Rasch analysis [24–26]. To date, few studies have used Rasch analysis to assess the psychometric properties of the PANSS [27–30]. Rasch analysis can provide evidence of anomalies with respect to two or more cultural groups in which an item can show differential item functioning (DIF). DIF can be used to establish whether a particular group show different scoring patterns within a rating scale [31–33]. DIF has been used to examine differences in rating scale scores with respect to translation, country, gender, ethnicity, age, and education level [34, 35].

The goal of this study was to examine the cross-cultural validity of the PANSS across six geo-cultural groups (Eastern Asia, India, Northern Europe, Russia & Ukraine, Southern Europe, and the United States of America) for data obtained from United States training videos (translated and subtitled for other languages). The study examines (1) measurement properties of the PANSS, namely dimensionality and score structure across cultures, (2) the validity of the PANSS across geo-cultural groups when assessing a patient from the United States, and (3) ways to enhance rater training based on cross-cultural differences in the PANSS.

## Methods

### Measures

The PANSS [13] is a 30-item scale used to evaluate the presence, absence and severity of Positive, Negative and General Psychopathology symptoms of schizophrenia. Each subscale contains individual items. The 30 items are arranged as seven positive symptom subscale items (P1 - P7), seven negative symptom subscale items (N1 - N7), and 16 general psychopathology symptom items (G1 - G16). All 30 items are rated on a 7-point scale (1 = absent; 7 = extreme). The PANSS was developed with a comprehensive anchor system to standardize administration, and improve the reliability of ratings. The potential range of scores on the Positive and Negative scales are 7 – 49, a score of 7 indicating no symptoms. The potential range of scores on the General Psychopathology Scale is 16 – 112.

The PANSS was scored by a clinician trained in psychiatric interview techniques, with experience working with the schizophrenia population (e.g., psychiatrists, mental healthcare professionals). A semi structured interview for the PANSS, the SCI-PANSS [36], was used as a guide during the interview.

Currently there are over 40 official language versions of the PANSS. This translation work has been carried out according to international guidelines, in co-operation between specific sponsors, together with translation agencies in the geo-cultural groups concerned. Translation standards for the PANSS followed internationally recognized guidelines with the objective to achieve semantic equivalence as outlined by Multi Health Systems (MHS Translation Policy, available at http://www.mhs.com/info.aspx?gr=mhs&prod=service&id=Translations). Semantic equivalence is concerned with the transfer of meaning across language.

### Rater training

For the data used in this study, each PANSS rater was required to obtain rater certification through ProPhase LLC, Rater Training Group, New York City, New York, and to achieve interrater reliability with an intraclass correlation coefficient = 0.80 with the “Expert consensus PANSS” scores (or Gold Score rating), in addition to other specified item and scale level criteria. Gold Score is described below. Only a Master’s level psychologist with one year experience working with schizophrenic patients and/or using clinical rating instruments, or a PhD level Psychologist, or Psychiatrist is eligible for PANSS rater certification. Rater training on the PANSS required the following steps:

1. First, a comprehensive, interactive, didactic tutorial was administered prior to the investigator meeting for the specified clinical trial. The tutorial was available at the Investigator’s Meeting, online, or on DVD or cassette for others. The tutorial included a comprehensive description of the PANSS and its associated items, after which the rater was required to view a video of a PANSS interview and rate each item.
2. Second, the rater was provided with feedback indicating the Gold Score rating of each item along with a justification for that score. The Gold Score rating was established by a group of four to five Psychiatrists or PhD level Psychologists who have administered the PANSS for ≥5 years. These individuals rated each interview independently. Scores for each of the interviews were combined and reviewed collectively in order to determine the Gold Score rating.
3. Once the rater completed the above steps with the qualifying scoring criteria, the rater was provisionally certified to complete the PANSS evaluations.

### Data

Data was obtained from ProPhase LLC Training Group (New York, NY) and are data from raters who scored PANSS training videos. The individuals depicted in the videos are actors who provided consent. The study data included PANSS scores from raters from the six geo-cultural groups who underwent training and rated one of 13 PANSS training videos. The symptoms presented in the 13 videos spanned the spectrum of psychopathology from absent to severe. Gold Scores for the 13 videos ranged from scores of 3 (Mild) to 6 (Severe) for Item P1 Delusions, 2 (Minimal) to 5 (Moderate Severe) for P2 Conceptual Disorganization, and 1 (Absent) to 5 (Moderate Severe) for the remaining Positive Symptom subscale items. For the Negative Symptom subscale items, scores ranged from 1 (Absent) to 5 (Moderate Severe) for Items N1 Blunted Affect, N4 (Passive Apathetic Social Withdrawal) and N6 Lack of Spontaneity and Flow of Conversation, with ranges of 1 (Absent) to 4 (Moderate) for Item N2 Emotional Withdrawal and N3 Poor Rapport, and 1 (Absent) to 6 (Severe) for Difficulty in Abstract Thinking. Scores on the 13 videos for the General Psychopathology also ranged from 1 (Absent) to 4 (Moderate) and 5 (Moderate Severe) for most items, with G9 Unusual Thought Content and G12 (Lack of Judgment and Insight) ranging from scores of 3 (Mild) to 6 (Severe). Data collection was conducted via a core data collection form that included completion of all 30 items of the PANSS. The form also contained information on one demographic variable of the raters which includes country of residency. The study recruitment took place from 2007 to 2011.

Data was obtained for 1,179 raters. Table 1 consists of sample characteristics and the distribution of countries per geo-cultural group. Data for African raters were not included in the analysis (i.e., 0.85% of total sample, n = 10; N = 1,179) due to inadequate sample size needed for comparison. One can note that the percentages of data that was removed for raters (from Africa (0.85%)) and for missing PANSS items (0.0%) are all reasonably small. These percentages point to the strong unlikelihood that analyses of these data would not be compromised by excluding these raters. It is not surprising to observe relatively no missing responses for the PANSS as scores on the instrument are incremental for training and raters are required to score each item for rater training and certification prior to the initiation of the study.

**Table 1:** **Sample characteristics and geo-cultural groupings**

Geo-cultural group	Countries	Total N
Northern Europe	Belgium, Czech Republic, Estonia, Aland (Finland), Germany, Lithuania, Netherlands, Poland, Slovakia, United Kingdom (UK), Hungary	126
Southern Europe	Bulgaria, Croatia, Israel, Romania, Serbia, Spain	162
Eastern Asia	Korea, Malaysia, Singapore, Taiwan, Japan	202
India	Republic of India	185
Russia & Ukraine	Russia, Ukraine	197
United States of America	United States of America (US)	297
Africa	South Africa	10
**TOTAL**		**1,179**

The study protocol was approved by Western Institutional Review Board, Olympia, WA for secondary analysis of existing data. Research involving human subjects (including human material or human data) that is reported in the manuscript was performed with the approval of an ethics committee (Western Institutional Review Board (WIRB) registered with OHRP/FDA; registration number is IRB00000533, parent organization number is IORG0000432.) in compliance with the Helsinki Declaration.

### Rasch analysis sample considerations

There are no established guidelines on the sample size required for Rasch and DIF analyses. The minimum number of respondents will depend on the type of method used, the distribution of the item response in the groups, and whether there are equal numbers in each group. Previous suggestions for minimum sample size for DIF analyses have usually been in the range of 100–200 per group [37, 38] to ensure adequate performance (>80% power). For the present study, an item shows DIF if there is not an equal probability of scoring consistently on a particular PANSS item [39] (p. 264).

### Selection of Geo-Cultural Groups

For this study, we assembled our data according to culture, with special attention to the presence and impact of clinical trials, and to the geographic residence of the raters. The resultant groups were defined prior to considering the amount of available data for each geo-cultural group.. An attempt was made to include raters who were likely to share more culturally within each group. The geo-cultural groups aim to gather the raters of a town, region, country, or continent on the basis of the realities and challenges of their society. Using geography in part to inform our cultural demarcations are not unproblematic or without limitations. Culture is necessarily social and is not strictly rooted in geography or lineage. However, the categories we elected for this study take into account geography as this was the criterion by which data were organized during rater training.

A few of our groups may appear unconventional at first glance. We separated India from other parts of Asia [38]. Table 1 presents the composition of the geo-cultural groupings. The groups are discursive and artificial constructs intended solely for the purpose of this study. No study of culture can involve all places and facets of life simultaneously and thus will reflect only generalities and approximations. For this reason, we were forced to overlook the multiple cultural subjectivities and hybridity [40], acculturation and appropriation [41], and fluidity that exist within and between the groups we constructed. The authors chose to keep the United States of America (US) as its own category since the scale is a cultural product of the US and was initially validated in this region.

As with any statistical analysis, if the categories were assembled differently (i.e., including or excluding certain groups, following a different organizing rationale) the analyses may have yielded slightly different results. However, the authors felt that there were enough similarities within the groupings: symptom expression and perception [42–44], clinical interview conduct [45], educational pedagogy and experience [46, 47], intellectual approach [48], ideas about individuality versus group identity [49], etc. to warrant our arrangement of data. An attempt also was made to group countries with related histories, educational and training programs and ethnicities under the assumption that the within-grouping differences are likely to be less than the between-grouping differences. Prevalence of English language fluency and exposure was not considered in our categorization. While local language training materials were made available in all cases (i.e., transcripts of patient videos) some training events included additional resources (i.e., translated didactic slides, on-site translators). The range of English-language comprehension varied greatly among raters as well between and within many of the categories. The variance caused by language itself or as a complex hybrid with cultural understanding and clinician experience with a measure or in clinical trials deserves more attention [50]. Therefore, it is recommended that a separate analysis of the effects of language on inter-rater reliability be conducted.

### Statistical methods

The Rasch measurement model assumes that the probability of a rater scoring an item is a function of the difference between the subject’s level of psychopathology and the level of psychopathology symptoms expressed by the item. Analyses conducted included assessment of the response format, overall model fit, individual item fit, differential item functioning (DIF), and dimensionality.

Inter-rater reliability: The internal consistency of the PANSS was tested through Cronbach α reliability coefficients whereas inter-rater reliability [51] was tested based on intra class correlation coefficient (ICC). The inter-rater reliability of the PANSS across all regions was assessed. We classified ICC above 0.75 as excellent agreement and below 0.4 as poor agreement [52].

Unidimensionality: DIF analyses assume that the underlying distribution of *θ* (the latent variable, i.e., psychopathology) is unidimensional [53], with all items measuring a single concept; for this reason, the PANSS subscales (Positive symptoms, Negative symptoms, and General Psychopathology) were used, as opposed to a total score. Dimensionality was examined by first conducting principal components analysis (PCA) assess unidimensionality as follows: (1) a PCA was conducted on the seven Positive Symptom items, (2) the eigenvalues for the first and second component produced by the PCA were compared, (3) if the first eigenvalue is about three times larger than the second one, dimensionality was assumed. Similar eigenvalue comparison was conducted for the seven items of the Negative Symptoms subscale and the 16 items of the General Psychopathology subscale [54] for methods of assessing unidimensionality using PCA). Suitability of the data for factor analysis was tested by Bartlett's Test of Sphericity [55] which should be significant, and the Kaiser-Meyer-Olkin (KMO) measure of sampling adequacy, which should be >0.6 [56].

Rasch Analysis: For each PANSS item a separate model was estimated using the response to that item as the dependent variable. The overall subscale score for the Positive symptoms, Negative symptoms, and General Psychopathology scale, and each cultural grouping, was the independent variables.

Two sets of Rasch analyses were conducted for each of the 30 items from the PANSS scale.

#### 1. Rasch analyses by geo-cultural grouping

To assess the measurement invariance of item calibrations across countries in the present study, the Rasch rating scale model was used [57]. The primary approach to addressing measurement invariance involves the study of group similarities and differences in patterns of responses to the items of the rating scale. Such analysis is concerned with the relative severity of individual test items for groups with dissimilar cultural or backgrounds. It seeks to identify items for which equally qualified raters from different cultural groups have different probabilities of endorsing a score of a particular item on the PANSS. To be used in different cultures, items must function the same way regardless of cultural differences. The Rasch model proposes that the responses to a set of items can be explained by a rater’s ability to assess symptoms and by the characteristics of the items. The Rasch rating scale model is based on the assumption that all PANSS subscale items have a shared structure for the response choices. The model provides estimates of the item locations that define the order of the items along the overall level of psychopathology.

Rasch analysis makes a calibration of items based on likelihood of endorsement (symptom severity). Inspection of item location is presented as average item calibrations (Δ Difficulty), goodness of fit (weighted mean square) and standard error (SE). The Rasch analysis was performed using jMetrik [58], where Δ Difficulty indicates that the lower the number (i.e., negative Δ), the less difficulty the rater has with that item. Taking into account the set order of the item calibrations based on ranking the Δ from smallest to largest, the adequacy of each item can be further evaluated by examining the pattern of easy and difficult items to rate based on culture (see Tables 2, 3 and 4b). When there is a good fit to the model (i.e., weighted mean square (WMS)), responses from individuals should correspond well with those predicted by the model. If the fit of most of the items is satisfactory, then the performance of the instrument is accurate. WMS fit statistics show the size of the randomness, i.e., the amount of distortion of the measurement system. Values less than 1.0 indicate observations are too predictable (redundancy, data overfit the model). Values greater than 1.0 indicate unpredictability (unmodeled noise, data underfit the model). Therefore a mean square of 1.5 indicates that there is 50% more randomness (i.e., noise) in the data than modeled. High mean-squares (WMS >2.0) were evaluated before low ones, because the average mean-square is usually forced to be near 1.0. Since, mean-square fit statistics average about 1.0, if an item was accepted with large mean-squares (low discrimination, WMS >2.0), then counter-balancing items with low mean-squares (high discrimination, WMS < 0.50) were also accepted.

**Table 2:** **Reliability estimates of raters across six regions**

Geo-cultural group	Positive symptoms	Negative symptoms	General psychopathology	Total PANSS score
Northern Europe				
*ICC (95% Confidence Interval)*	0.987 (0.948, 0.996)	0.928 (0.831, 0.985)	0.926 (0.929, 0.984)	0.973 (0.958, 0.985)
Southern Europe				
*ICC (95% Confidence Interval)*	0.991 (0.979, 0.998)	0.967 (0.921, 0.993)	0.982 (0.968, 0.993)	0.987 (0.980, 0.993)
Russia & Ukraine				
*ICC (95% Confidence Interval)*	0.987 (0.969, 0.997)	0.975 (0.939, 0.995)	0.978 (0.960, 0.991)	0.983 (0.975, 0.990)
India				
*ICC (95% Confidence Interval)*	0.986 (0.966, 0.997)	0.955 (0.895, 0.991)	0.981 (0.965, 0.993)	0.984 (0.975, 0.991)
Eastern Asia				
*ICC (95% Confidence Interval)*	0.987 (0.969, 0.997)	0.953 (0.888, 0.990)	0.980 (0.963, 0.992)	0.981 (0.970, 0.989)
United States of America				
*ICC (95% Confidence Interval)*	0.992 (0.980, 0.998)	0.965 (0.916, 0.993)	0.988 (0.978, 0.995)	0.990 (0.983, 0.994)

**Table 3:** **Comparison between different geo-cultural groups of PANSS item Rasch rating scale item difficulty (Δ) and goodness of fit (weighted mean square WMS values: positive symptoms, negative symptoms, general psychopathology**

PANSS items	Northern Europe			Southern Europe			India			Eastern Asia			Russia & Ukraine			USA		
Positive Symptoms	Difficulty (Δ)	WMS	SE	Difficulty (Δ)	WMS	SE	Difficulty (Δ)	WMS	SE	Difficulty (Δ)	WMS	SE	Difficulty (Δ)	WMS	SE	Difficulty (Δ)	WMS	SE
P1.	−0.68	3.05	0.07	−0.79	2.86	0.05	−0.60	2.22	0.05	−0.52	1.49	0.04	−0.44	2.84	0.05	−0.38	1.34	0.06
P2.	−0.26	2.26	0.06	−0.30	1.60	0.05	−0.13	2.18	0.05	−0.28	0.78	0.05	−0.22	1.67	0.06	−0.14	1.65	0.04
P3.	−0.80	2.17	0.07	- 0.81	2.10	0.05	−0.79	0.94	0.10	−0.63	0.81	0.04	−0.63	0.81	0.04	−0.72	1.43	0.04
P4.	0.30	2.15	0.07	0.60	1.55	0.04	0.54	1.96	0.06	0.69	1.18	0.06	0.69	1.18	0.06	0.53	1.62	0.04
P5.	−0.27	2.41	0.06	0.51	2.00	0.04	0.13	2.34	0.05	0.50	2.40	0.05	−0.54	2.03	0.05	−0.08	1.89	0.04
P6.	−0.58	2.62	0.07	−0.69	1.89	0.06	−0.64	2.06	0.05	−0.69	1.48	0.05	−0.66	1.84	0.06	−0.93	1.90	−0.93
P7.	0.11	1.89	0.06	0.21	1.44	0.05	−0.09	1.84	0.05	0.03	0.75	0.04	0.23	1.39	0.06	0.12	1.59	0.12
Negative Symptoms	Difficulty (Δ)	WMS	SE	Difficulty (Δ)	WMS	SE	Difficulty (Δ)	WMS	SE	Difficulty (Δ)	WMS	SE	Difficulty (Δ)	WMS	SE	Difficulty (Δ)	WMS	SE
N1.	−0.23	2.88	0.06	0.30	2.81	0.06	0.10	0.60	0.07	0.28	1.93	0.06	0.22	2.01	0.05	−0.23	2.88	0.06
N2.	−0.25	1.61	0.06	−0.30	1.60	0.06	−0.38	1.47	0.05	−0.36	1.11	0.04	−0.22	1.57	0.05	−0.24	1.61	0.06
N3.	0.01	2.09	0.06	0.09	2.00	0.05	−0.26	1.00	0.05	0.08	0.90	0.05	0.10	2.11	0.05	0.01	2.09	0.06
N4.	−0.18	1.68	0.06	−0.20	1.58	0.05	−0.19	1.30	0.05	−0.16	1.01	0.04	−0.13	1.67	0.06	−0.18	1.68	0.06
N5.	−0.55	2.03	0.07	0.20	2.01	0.06	−0.56	1.34	0.05	0.15	0.74	0.06	0.16	2.02	0.06	−0.55	2.03	0.07
N6.	−0.28	1.84	0.06	−0.10	1.80	0.05	−0.52	1.16	0.05	−0.19	0.82	0.04	−0.55	1.79	0.06	−0.28	1.84	0.06
N7.	0.21	1.46	0.06	0.43	1.41	0.06	0.69	1.22	0.08	0.29	0.84	0.05	0.60	1.31	0.07	0.21	1.46	0.06
General Psychopathology	Difficulty (Δ)	WMS	SE	Difficulty (Δ)	WMS	SE	Difficulty (Δ)	WMS	SE	Difficulty (Δ)	WMS	SE	Difficulty (Δ)	WMS	SE	Difficulty (Δ)	WMS	SE
G1.	0.22	1.99	0.06	0.41	1.18	0.07	0.63	1.51	0.06	0.55	0.80	0.06	0.40	1.10	0.06	0.80	1.78	0.05
G2.	0.10	1.58	0.10	0.15	1.05	0.09	0.01	1.86	0.05	−0.25	1.25	0.07	0.15	1.04	0.09	−0.01	1.02	0.05
G3.	0.72	2.23	0.08	1.00	2.01	0.07	1.38	1.82	0.09	0.81	1.38	0.07	1.41	1.05	0.05	0.93	2.36	0.06
G4.	0.29	1.71	0.07	0.39	1.00	0.05	0.46	1.47	0.06	0.29	0.62	0.05	0.57	1.04	0.05	0.39	0.96	0.04
G5.	0.69	1.40	0.08	0.23	1.14	0.07	0.86	1.21	0.07	1.12	1.25	0.08	1.11	1.24	0.07	0.84	1.44	0.05
G6.	−0.06	2.66	0.06	0.90	1.06	0.06	0.37	2.59	0.05	0.66	1.67	0.06	0.97	1.32	0.06	0.34	0.76	0.05
G7.	0.40	1.55	0.07	0.41	1.50	0.06	0.04	1.26	0.05	0.01	0.89	0.04	0.47	1.35	0.05	0.27	0.64	0.04
G8.	0.23	1.63	0.06	0.79	0.74	0.09	0.10	1.64	0.05	0.16	0.71	0.05	0.77	0.76	0.05	0.13	1.09	0.04
G9.	−0.34	2.77	0.06	−0.55	1.09	0.10	−0.08	2.00	0.05	−0.46	0.88	0.07	−0.34	1.23	0.09	−0.16	1.55	0.04
G10	0.41	0.71	0.08	0.06	0.77	0.07	0.22	1.27	0.05	0.20	1.32	0.05	0.21	1.22	0.05	0.69	1.42	0.05
G11.	0.27	1.39	0.07	0.01	0.82	0.08	0.17	1.46	0.05	0.03	0.77	0.04	0.22	1.02	0.07	0.31	1.10	0.04
G12.	−0.51	0.50	0.09	−0.48	0.79	0.07	−0.75	1.34	0.05	−0.53	1.16	0.05	−0.50	0.99	0.07	−0.27	1.39	0.04
G13.	0.12	1.87	0.06	0.24	1.80	0.05	0.04	1.61	0.05	−0.17	0.85	0.04	0.26	0.88	0.05	0.20	0.87	0.04
G14.	0.98	3.36	0.09	0.90	2.98	0.06	0.58	2.43	0.06	0.40	0.97	0.05	0.90	2.07	0.06	0.84	1.62	0.05
G15.	0.31	1.66	0.07	0.06	0.75	0.08	0.01	1.66	0.05	0.15	0.66	0.05	0.63	1.60	0.07	0.22	0.95	0.04
G16.	−0.19	2.16	0.06	0.55	1.23	0.06	−0.29	2.03	0.05	−0.27	1.20	0.09	0.60	1.45	0.07	−0.55	2.10	0.04

*WMS*: Weighted Mean Square; *UMS*: Unweighted Mean Square. *SE* = Standard Error.

**Table 4:** **Differential item functioning positive and negative symptoms: reference group = USA vs. focal group = Northern European, Southern Europe and Russia & Ukraine**

Northern Europe						Southern Europe					Russia & Ukraine					USA
Item	Chi-sq	p-value	E.S. (95% C.I.)	Class	Northern Europe Mean	Chi-sq	p-value	E.S. (95% C.I.)	Class	Southern Europe Mean	Chi-sq	p-value	E.S. (95% C.I.)	Class	Russo Europe	USA Mean
P1	0.79	0.38	−0.02 (−0.21;0.17)	A	4.60 (1.06)	27.73	< 0.001	−0.56 (−0.76;-0.35)	B-	3.66 (0.75)*	6.06	0.01	−0.31 (−0.50;-0.12)	BB-	3.86 (0.84)	4.29 (1.05)
P2	4.16	0.04	0.22 (−0.04;0.48)	A	3.97 (0.99)	26.9	< 0.001	0.83 (0.56;1.10)	C+	4.05 (1.34)*	6.58	0.01	0.34 (0.12;0.55)	BB+	3.56 (0.77)	3.42 (1.34)
P3	3.93	0.05	0.12 (−0.03;0.27)	A	4.79 (0.68)	4.48	0.03	0.20 (0.03;0.38)	A	4.24 (0.82)	8.68	< 0.001	0.24 (0.09;0.38)	AA	4.40 (0.84)*	4.33 (0.96)
P4	0.84	0.36	−0.07 (−0.26;0.12)	A	3.11 (1.13)	2.55	0.11	−0.18 (−0.35;-0.01)	A	2.07 (1.25)	0.42	0.52	−0.04 (−0.21;0.12)	AA	2.40 (1.24)	2.70 (1.80)
P5	0.4	0.53	0.12 (−0.06;0.31)	A	3.98 (1.43)	40.17	< 0.001	−0.63 (−0.83;-0.42)	C-	2.10 (1.49)*	2.2	0.14	−0.04 (−0.23;0.15)	AA	2.87 (1.40)	3.34 (1.33)
P6	15.42	< 0.001	−0.33 (−0.51;-0.15)	B-	4.46 (0.88)*	12.95	< 0.001	−0.39 (−0.59;-0.20)	B-	1.09 (0.81)*	27.12	< 0.001	−0.59 (−0.80;-0.39)	BB-	3.88 (1.20)*	4.64 (1.02)
P7	0.3	0.59	−0.04 (−0.25;0.18)	A	3.39 (0.93)	56.93	< 0.001	0.72 (0.53;0.91)	C+	3.32 (1.03)*	14.33	< 0.001	0.41 (0.22;0.60)	BB+	3.21 (1.13)*	3.05 (1.26)
N1	34.81	<0.001	−0.56 (−0.76;-0.36)	BB-	3.91 (1.30)*	1.89	0.17	−0.09 (−0.24;0.05)	AA	4.46 (1.29)	0.4	0.53	−0.03 (−0.26;0.19)	AA	4.10 (1.09)	4.01 (1.67)
N2	0.6	0.44	0.07 (−0.05;0.20)	AA	3.94 (0.55)	0.46	0.5	0.03 (−0.07;0.12)	AA	4.02 (0.61)	0.48	0.49	−0.08 (−0.22;0.06)	AA	3.63 (0.76)	3.85 (0.79)
N3	1	0.32	0.10 (−0.08;0.27)	AA	3.56 (1.30)	30.61	< 0.001	0.44 (0.29;0.59)	BB+	4.04 (1.23)*	7.54	0.01	0.20 (0.05;0.34)	AA	3.24 (0.85)	3.26 (1.51)
N4	0.01	0.93	0.01 (−0.16;0.17)	AA	3.84 (0.99)	37.25	< 0.001	−0.55 (−0.70;-0.39)	BB-	3.49 (0.84)*	2.27	0.13	0.02 (−0.15;0.19)	AA	3.52 (0.87)	3.74 (1.23)
N5	0.03	0.86	−0.03 (−0.23;0.18)	AA	4.41 (1.14)	15.71	< 0.001	−0.36 (−0.55;-0.18)	BB-	4.04 (1.07)*	7.78	0.01	−0.24 (−0.44;-0.04)	AA	3.94 (0.94)	4.16 (1.32)
N6	0.93	0.33	0.06 (−0.12;0.24)	AA	3.99 (1.39)	20.58	< 0.001	0.36 (0.20;0.51)	BB+	4.39 (1.30)*	9.07	< 0.001	0.02 (−0.17;0.21)	AA	3.54 (1.08)*	3.52 (1.73)
N7	10.44	<0.001	0.35 (0.15;0.56)	BB+	3.25 (0.86)*	2.44	0.12	0.18 (−0.00;0.37)	AA	3.21 (0.99)	0.33	0.57	0.11 (−0.06;0.29)	AA	2.89 (0.77)	2.17 (1.15)

* Bonferroni Corrected p <0 .0017; E.S.: Effect Size; Chi-sq: Chi Square.

#### 2. DIF analyses by geo-cultural grouping

Based on the results of Rasch analyses different approaches can be taken to account for weaknesses in the scoring properties of the PANSS post-hoc. The Mantel-Haenszel statistic is commonly used in studies of DIF, because it makes meaningful comparisons of item performance for different geographical groups, by comparing raters of similar cultural backgrounds, instead of comparing overall group performance on an item. In a typical differential item functioning (DIF) analysis, a significance test is conducted for each item. As the scale consists of multiple items, such multiple testing may increase the possibility of making a Type I error at least once. Type I error rate can be affected by several factors, including multiple testing. For DIF of the 30 item PANSS the expectation is that 2 item response strings have a probability of p ≤.05 according with the Rasch model. α is the Type I error for a single test (incorrectly rejecting a true null hypothesis). So, when the data fit the model, the probability of a correct finding for one item is (1-α), and for n items, (1-α)^n^. Consequently the Type I error for n independent items is 1-(1-&alpha)^n^. Thus, the level for each single test is α/n. So that for a finding of p ≤ .05 to be found for 30 items, then at least one item would need to be reported with p ≤ .0017 on a single item test for the hypothesis that "the entire set of items fits the Rasch model" to be rejected.

As the PANSS was developed in the US and the rater training was conducted by a training facility in the US, the authors chose to compare each geo-cultural group to the US. Additionally, raters in similar geo-cultural groups were compared (e.g., Northern European raters vs. Southern European raters, Eastern Asian raters (will here forth be referred to as Asia or Asian) vs. Indian raters, Northern European raters vs. Russia & Ukraine raters). The Mantel-Haenszel procedure is performed in jMetrik and produces effect size computation and Educational Testing Services (ETS) DIF classifications as follows:

- A. = Negligible DIF
- B. = Slight to Moderate DIF
- C. = Moderate to Large DIF

Operational items categorized as C are carefully reviewed to determine whether there is a plausible reason why any aspect of that item may be unfairly related to group membership, and may or may not be retained on the test.

Additionally, each category A, B or C is scored as either – or + where,

- : Favors reference group (indicating the item is easier to score for this group, than the comparison group)

+ : Favors focal group (indicating the item is easier to score for this group, than the comparison group)

## Results

### Reliability

Reliability was assessed for each of the six geo-cultural groups and results are as follows: Cronbach alpha (α) and Intra Class Coefficients (ICC) for all groups were excellent and Average Measures ICCs were significant at p < 0.001 for all groups (Northern Europe = Cronbach α = 0.977, ICC = 0.973 (95% CI = 0.958, 0.985); Southern Europe = Cronbach α = 0.989, ICC = 0.987 (95% CI = 0.980, 0.993); India = Cronbach α = 0.987, ICC = 0.984 (95% CI = 0.975, 0.991); Asia = Cronbach α = 0.984, ICC = 0.981 (95% CI = 0.970, 0.989); Russia & Ukraine = Cronbach α = 0.987, ICC = 0.983 (95% CI = 0.975, 0.990); United States of America = Cronbach α = 0.991, ICC = 0.990 (95% CI = 0.983, 0.994) (see Table 2).

Reliability for subscale measures also show excellent reliability across all three subscales for each of the six geo-cultural groups.

### Assessment of unidimensionality

Principal Components Analysis (PCA) without rotation revealed one component with an eigenvalue greater than one for the Positive Symptoms subscale, one component with an eigenvalue greater than one for the Negative Symptoms subscale and four components with an eigenvalue greater than one for the General Psychopathology subscale. Bartlett's Test of Sphericity was significant (p < .001) for all three subscales and the Kaiser-Meyer-Olkin (KMO) measure of sampling adequacy produced values of 0.790, 0.877, and 0.821 for the Positive, Negative and General Psychopathology subscales, respectively. Using the criteria to assess unidimensionality of the eigenvalue for the first component being three times larger than the second component, the Positive and Negative Symptoms subscales indicate unidimensionality while the General Psychopathology subscale shows an eigenvalue on the second component of only 1.230 times larger than the first component. Although the General Psychopathology subscale was not unidimensional, basic steps for validating items were met, i.e., intraclass correlations were all ≥ 0.90, and the items of the General Psychopathology subscale was evenly distributed and linear.

### Rasch analysis

Most items showed high mean squares (WMS > 2.0 or low discrimination). Poor fit does not mean that the Rasch measures (parameter estimates) aren't additive (appropriate). The Rasch model forces its estimates to be additive. So a WMS > 2.0 suggests a deviation from unidimensionality in the data, not in the measures. Therefore, values greater than 2.0 (see Table 3) indicate unpredictability (unmodeled noise, model underfit). Items with high WMS were examined first (to assess which items may have been influenced by outliers), and temporarily removed from the analysis, before investigating the items with low WMS, until WMS values were closer to 1.0.

#### Positive symptoms

Average item calibrations and goodness of fit values for each PANSS Positive subscale item for the 6 geo-cultural groups are presented in Table 3. Lower item calibration reflects items easy to endorse, in which raters often showed less difficulty scoring; higher item calibration reflects items more difficultly scoring. Items varied in severity from −0.93 [item P6. Suspiciousness/persecution (USA) to 0.69 item P4. Excitement (Asia)]. Item P3 (Hallucinatory Behavior) was the easiest item to score for all geo-cultural groups ranging from Russia & Ukraine (Δ = −0.82) to Asia (Δ = −0.63), followed by item P6 (Suspiciousness/Persecution), which ranged from United States (Δ = −0.93) to Northern Europe ((Δ = −0.58). All geo-cultural groups found item P4 (Excitement) the most difficult to score across all items. With difficulty levels ranging from Δ = 0.69 (Asia) to Δ = 0.30 (Northern Europe). P5 (Grandiosity) was the most difficult for Southern Europe (Δ = 0.51) and Asia (Δ = 0.50). Overall, the goodness-of-fit of the PANSS Positive items was satisfactory across all geo-cultural groups.

#### Negative symptoms

Average item calibrations and goodness of fit values for each PANSS Negative subscale item for the 6 geo-cultural groups are presented in Table 3. Lower item calibration reflects items easy to endorse, in which raters often showed less difficulty scoring; higher item calibration reflects items more difficultly scoring. Items varied in severity from −0.56 [item N5. Difficulty in Abstract Thinking (India)] to 0.69 [item N7. Stereotyped Thinking (India)]. Item N5 (Difficulty in Abstract Thinking) was the easiest in Northern Europe, USA (Δ = −0.55 respectively), and India (Δ = −0.56). For the remaining items, the easiest item to rate was N2 (Emotional Withdrawal) for Southern Europe (Δ = −0.30) and Asia (Δ = −0.36). The easiest Negative symptom item to score for Russia & Ukraine is N6 (Lack of Spontaneity and Flow of Conversation), Δ = −0.55. The most difficult item to score for all groups is N7 (Stereotyped Thinking) with India showing the most difficulty Δ = 0.69, and Northern Europe and the United States of America showing the least difficulty Δ = 0.21, each. The second most difficult item for raters to score was N1 (Blunted Affect) for most groups including Southern Europe (Δ = 0.30), Asia (Δ = 0.28), Russia & Ukraine (Δ = 0.22) and India (Δ = 0.10). Russia & Ukraine also had difficulties scoring N5 (Difficulty in Abstract Thinking) Δ = 0.16. Overall, the goodness-of-fit of the PANSS Positive items was satisfactory across all geo-cultural groups.

#### General psychopathology symptoms

Average item calibrations and goodness of fit values for each PANSS General Psychopathology subscale item for the 6 geo-cultural groups are presented in Table 3. Lower item calibration reflects items easy to endorse, in which raters often showed less difficulty scoring; higher item calibration reflects items more difficultly scoring. Items varied in severity from −0.75 [G12 Lack of Judgment and Insight (India)] to 1.41 [item G3 Guilt Feelings (Russia & Ukraine)]. All geo-cultural groups had item G12 (Lack of Judgment and Insight) as the least difficult item to score with Indian raters having the least difficulty (Δ = −0.75), along with item G2 (Anxiety) with Asian raters having the least difficulty (Δ = −0.25). Northern European raters had the least difficulty with item G6. Depression (Δ = −0.06). Other items which were easier to score included G16 (Active Social Avoidance) for United States raters (Δ = −0.55), Indian raters (Δ = −0.29), Asian raters (Δ = −0.27). However, Southern European raters, and Russian & Ukrainian raters had item G16 among the most difficult to score, with Δ = 0.55 and Δ = 0.60, respectively.

The most difficult item for raters to score for all groups is G4 (Tension) with difficulty levels ranging from Δ = 1.38 (India) to Δ = 0.72, and item G3 (Guilt Feelings) for Russia & Ukraine raters (Δ = 1.41). Also, G10 (Disorientation) for Northern Europe (Δ = 0.41), India (Δ = 0.22), Asia (Δ = 0.20), and the United States of America (Δ = 0.69) were difficult to score. Other geo-cultural groups also showed difficulties rating this item (see Table 2). Also, raters from Asia had difficulties scoring item G5 (Mannerisms and Posturing), Δ = 0.81). Raters from Southern Europe, India, Asia, Russia & Ukraine and the United States of America also had difficulties scoring item G14 (Poor Impulse Control) with item difficulty ranging from Δ = 0.90 (Southern Europe) to Δ = 0.40 (Asia). Russia & Ukraine also had significant difficulty with item G15 (Preoccupation). Overall, the goodness-of-fit of the PANSS Positive items was satisfactory across all geo-cultural groups.

### Differential item functioning analysis

#### Positive symptoms

Northern Europe: Significant DIF was found for items P6. Suspiciousness/Persecution (Chi Square = 15.42, p < 0.001) for USA and Northern Europe. P6 Suspiciousness/Persecution shows Slight to Moderate DIF (Class B) favoring the US (or reference group) (see Table 4).

Southern Europe: Significant DIF was found for items P1. Delusions, P2. Conceptual Disorganization, P5. Grandiosity, P6. Suspiciousness/Persecution and P7. Hostility for Southern European raters compared to USA raters. Of the significant items, P1. Delusions, and P6. Suspiciousness/Persecution shows slight to moderate DIF (Class B) favoring the United States (reference group), whilst P2. Conceptual Disorganization, and P7. Hostility shows Moderate to Large DIF (Class C) favoring Southern Europe. Moderate to large DIF (Class C) is also seen for P5 Grandiosity favoring the US (see Table 4).

Russia & Ukraine: Significant DIF was found for items P3. Hallucinatory Behavior, P6. Suspiciousness/Persecution, and P7. Hostility for Russia & Ukraine raters compared to USA raters. Of the significant items, P6. Suspiciousness/Persecution showed Slight to Moderate DIF (Class B) favoring the USA raters, whilst P7. Hostility showed slight to moderate DIF favoring Russia & Ukraine. Negligible DIF (Class A) was observed for P3. Hallucinatory Behavior (see Table 4).

India: Significant DIF was found for items P3. Hallucinatory Behavior, P6. Suspiciousness/Persecution, and P7. Hostility for Indian raters compared to USA raters. Of the significant items, P6. Suspiciousness/Persecution shows slight to moderate DIF (Class B) favoring the United States (reference group), whilst P7. Hostility shows slight to moderate DIF (Class B) favoring India. Negligible DIF (Class A) was observed for P3. Hallucinatory Behavior (see Table 5).

**Table 5:** **Differential item functioning positive and negative symptoms: reference group = USA vs. focal group = India and East & Maritime Asia**

	India					Eastern Asia					
Item	Chi-sq	p-value	E.S. (95% C.I.)	Class	India	Chi-sq	p-value	E.S. (95% C.I.)	Class	Eastern Asia	USA Mean
P1	2.4	0.12	−0.15 (−0.32;0.02)	A	4.19 (0.99)	7.55	0.01	−0.32 (−0.52;-0.13)	BB-	3.89 (1.01)	4.29 (1.05)
P2	0.01	0.94	0.06 (−0.17;0.29)	A	3.46 (1.32)	1.72	0.19	0.29 (0.06;0.52)	AA	3.52 (1.81)	3.42 (1.34)
P3	8.78	< 0.001	0.25 (0.10;0.40)	A	4.53 (0.96)*	0.01	0.94	−0.03 (−0.23;0.17)	AA	4.06 (1.18)	4.33 (0.96)
P4	0.39	0.53	−0.07 (−0.22;0.07)	A	2.55 (1.18)	6.78	0.01	0.19 ( 0.03;0.36)	AA	2.43 (1.12)	2.70 (1.80)
P5	2.62	0.11	−0.16 (−0.34;0.02)	A	3.08 (1.70)	7.27	0.01	−0.28 (−0.49;-0.06)	AA	2.47 (1.65)	3.34 (1.33)
P6	19.93	< 0.001	−0.37 (−0.53;-0.21)	B-	4.25 (0.78)*	5.77	0.02	−0.29 (−0.46;-0.11)	AA	4.16 (0.87)	4.64 (1.02)
P7	22.91	< 0.001	0.44 (0.26;0.61)	B+	3.39 (0.86)*	14.21	< 0.001	0.43 (0.23;0.64)	BB+	3.07 (1.06)*	3.05 (1.26)
N1	7.86	0.01	−0.20 (−0.34;-0.06)	AA	4.05 (1.50)	0.09	0.76	0.09 (−0.06;0.24)	AA	3.85 (1.66)	4.01 (1.67)
N2	0.02	0.9	0.01 (−0.09;0.12)	AA	3.85 (0.77)	0.59	0.44	−0.10 (−0.21;0.01)	AA	3.65 (0.69)	3.85 (0.79)
N3	15.16	< 0.001	0.31 (0.17;0.45)	BB+	3.66 (1.41)*	3.45	0.06	0.16 (0.01;0.30)	AA	3.00 (1.29)	3.26 (1.51)
N4	8.98	< 0.001	−0.23 (−0.37;-0.09)	AA	3.55 (1.01)*	4.91	0.03	−0.16 (−0.29;-0.04)	AA	3.34 (0.77)	3.74 (1.23)
N5	2	0.16	−0.15 (−0.33;0.03)	AA	4.14 (1.23)	1.57	0.21	−0.12 (−0.31;0.06)	AA	3.83 (1.41)	4.16 (1.32)
N6	13.42	< 0.001	0.29 (0.13;0.44)	AA	4.06 (1.62)*	0	0.98	−0.01 (−0.16;0.14)	AA	3.24 (1.33)	3.52 (1.73)
N7	0.02	0.88	−0.03 (−0.22;0.17)	AA	2.89 (1.21)	2.81	0.09	0.15 (−0.02;0.33)	AA	2.88 (1.83)	2.17 (1.15)

* Bonferroni Corrected p <0 .0017; E.S.: Effect Size; Chi-sq: Chi Square.

Asia: Significant DIF was found for P7. Hostility for Asian raters compared to USA raters. P7. Hostility was showed Slight to Moderate DIF (Class B) favoring Asian raters (see Table 5).

#### Negative symptoms

Northern Europe: Significant DIF was found for items N1. Blunted Affect and N7. Stereotyped Thinking for Northern European raters compared to US raters. Of the significant items N1. Blunted Affect showed slight to moderate DIF (Class B) favoring USA and N7. Stereotyped Thinking showed slight to moderate DIF (Class B) favoring Northern Europe (see Table 4).

Southern Europe: Significant DIF was found for N3. Poor Rapport, N4. Passive Apathetic Social Withdrawal, N5. Difficulty in Abstract Thinking, and N6. Lack of Spontaneity/Flow of Conversation for Southern European raters compared to US raters. N4. Passive Apathetic Social Withdrawal and N5. Difficulty in Abstract Thinking showed slight to moderate DIF (Class B) favoring US, and N3. Poor Rapport and N6. Lack of Spontaneity/Flow of Conversation showed slight to moderate DIF (Class B) favoring Southern Europe (see Table 4).

Russia & Ukraine: Significant DIF was found for N6. Lack of Spontaneity and Flow of Conversation; Negligible DIF (Class A) was observed for scores obtained for raters from Russia & Ukraine compared to US raters (see Table 4).

India: Significant DIF was found for items N3. Poor Rapport, N4. Passive Apathetic Social Withdrawal, and N6. Lack of Spontaneity and Flow of Conversation for Indian raters compared to USA raters. Of the significant items, only N3. Poor Rapport showed slight to moderate DIF (Class B) Indian Raters. Negligible DIF (Class A) was observed for N4. Passive Apathetic Social Withdrawal, and N6. Lack of Spontaneity and Flow of Conversation (see Table 5).

Asia: No significant DIF was found for Asian raters compared to US raters (see Table 5).

#### General psychopathology

Northern Europe: Significant slight to moderate DIF (Class B) DIF was observed for G2. Anxiety, G3. Guilt Feelings, G6. Depression, G7. Motor Retardation, G9. Unusual Thought Content, and G16. Active Social Avoidance. G2. Anxiety, G3. Guilt Feelings, G6. Depression, G7. Motor Retardation, and G16. Active Social Avoidance favored the USA raters; G3. Guilt Feelings and G9. Unusual Thought Content favored the Northern Europe raters. Items G1. Somatic Concern and G10. Disorientation showed moderate to severe DIF (Class C) both favoring Northern Europe raters (see Table 6).

**Table 6:** **Differential item functioning positive and negative symptoms: reference group = USA vs. focal group = Northern Europe, Southern Europe, Russia & Ukraine, India, Eastern Asia**

Northern Europe					Southern Europe				Russia & Ukraine				India				Eastern Asia			
Item	Chi-sq	p-value	E.S. (95% C.I.)	Class	Chi-sq	p-value	E.S. (95% C.I.)	Class	Chi-sq	p-value	E.S. (95% C.I.)	Class	Chi-sq	p-value	E.S. (95% C.I.)	Class	Chi-sq	p-value	E.S. (95% C.I.)	Class
G1	17.54*	<0.001	0.73 (0.40;1.06)	CC+	1.74	0.19	0.19 (−0.09;0.48)	AA	12.94*	< 0.001	0.46 (0.23;0.69)	BB+	1.07	0.3	0.15 (−0.09;0.39)	AA	12.67*	<0.001	0.50 (0.24;0.76)	BB+
G2	21.95*	<0.001	−0.55 (−0.79;-0.30)	BB-	57.91*	<0.001	−0.77 (−0.98;-0.56)	CC-	12.2*	< 0.001	−0.35 (−0.55;-0.16)	BB-	36.56*	<0.001	−0.66 (−0.88;-0.45)	CC-	15.96*	<0.001	−0.47 (−0.69;-0.24)	BB-
G3	3.85*	<0.001	0.38 (0.04;0.72)	BB+	19.01*	<0.001	−0.62 (−0.91;-0.34)	CC-	8.58*	< 0.001	−0.39 (−0.64;-0.14)	BB-	13.56*	<0.001	−0.49 (−0.75;-0.24)	BB-	0.65	0.42	−0.08 (−0.33;0.17)	AA
G4	0.13	0.72	0.08 (−0.15;0.31)	AA	4.52	0.03	−0.21 (−0.38;-0.03)	AA	3.84	0.05	−0.15 (−0.34;0.03)	AA	5.85	0.02	−0.26 (−0.42;-0.10)	AA	0.96	0.33	−0.08 (−0.25;0.09)	AA
G5	0.29	0.59	−0.10 (−0.37;0.17)	AA	7.32	0.01	−0.35 (−0.57;-0.14)	BB-	4.11	0.04	0.07 (−0.13;0.27)	AA	2.01	0.16	−0.15 (−0.36;0.06)	AA	21.1*	<0.001	−0.43 (−0.63;-0.23)	BB-
G6	6.72	0.01	−0.42 (−0.71;-0.14)	BB-	96.09*	<0.001	−1.56 (−1.88;-1.24)	CC-	12.62*	< 0.001	−0.44 (−0.68;-0.20)	BB-	43.1*	< 0.001	−0.96 (−1.24;-0.67)	CC-	101.6*	<0.001	−1.42 (−1.70;-1.13)	CC-
G7	7.99*	<0.001	−0.35 (−0.56;-0.14)	BB-	14.83*	<0.001	0.33 (0.14;0.52)	BB+	0.05	0.82	−0.08 (−0.30;0.14)	AA	9.06*	< 0.001	0.28 (0.10;0.46)	AA	4.75	0.03	0.21 (0.01;0.40)	AA
G8	0.38	0.54	−0.06 (−0.27;0.14)	AA	0.24	0.63	0.01 (−0.19;0.20)	AA	19.24*	< 0.001	−0.40 (−0.61;-0.19)	BB-	1.22	0.27	0.15 (−0.03;0.33)	AA	1.95	0.16	−0.12 (−0.30;0.06)	AA
G9	12.25*	<0.001	0.47 (0.19;0.74)	BB+	0.45	0.5	−0.09 (−0.29;0.11)	AA	4.92	0.03	−0.30 (−0.49;-0.10)	AA	0.42	0.52	−0.07 (−0.29;0.15)	AA	24.13*	<0.001	0.55 (0.33;0.76)	BB+
G10	12.43*	<0.001	0.62 (0.32;0.93)	CC+	51.21*	<0.001	0.81 (0.59;1.03)	CC+	24.08*	< 0.001	0.54 (0.35;0.72)	BB+	35.63*	< 0.001	0.63 (0.42;0.84)	CC+	21.37*	<0.001	0.47 (0.25;0.69)	BB+
G11	0.03	0.86	0.00 (−0.20;0.20)	AA	38.47*	<0.001	0.64 (0.43;0.86)	CC+	0.37	0.54	−0.02 (−0.19;0.15)	AA	1.68	0.19	0.13 (−0.05;0.31)	AA	12.14*	<0.001	0.30 (0.12;0.48)	BB+
G12	3.91	0.05	0.24 (0.01;0.48)	AA	16.84*	<0.001	0.44 (0.21;0.66)	BB+	8.07*	< 0.001	0.28 (0.08;0.49)	AA	44.49*	< 0.001	0.76 (0.54;0.98)	CC+	17.92*	<0.001	0.40 (0.19;0.62)	BB+
G13	0.03	0.86	−0.12 (−0.32;0.08)	AA	46.14*	<0.001	0.61 (0.43;0.79)	CC+	19.82*	< 0.001	0.32 (0.14;0.50)	BB+	4.2	0.04	0.16 (−0.02;0.35)	AA	16.89*	<0.001	0.34 (0.16;0.52)	BB+
G14	1.73	0.19	−0.22 (−0.49;0.05)	AA	27.31*	<0.001	0.60 (0.39;0.81)	CC+	20.86*	< 0.001	0.48 (0.29;0.67)	BB+	8.34*	< 0.001	0.31 (0.10;0.53)	BB+	20.34*	<0.001	0.40 (0.21;0.58)	BB+
G15	6.35	0.01	−0.29 (−0.54;-0.04)	AA	8.39*	<0.001	0.27 (0.08;0.46)	AA	2.91	0.09	0.19 (0.01;0.37)	AA	8.29*	< 0.001	0.28 (0.08;0.48)	AA	0.04	0.84	−0.01 (−0.18;0.17)	AA
G16	7.97*	<0.001	−0.40 (−0.69;-0.11)	BB-	9.45*	<0.001	−0.31 (−0.51;-0.10)	BB-	8.93*	< 0.001	−0.21 (−0.39;-0.03)	AA	6.01	0.01	−0.27 (−0.50;-0.04)	AA	31.17*	<0.001	−0.56 (−0.77;-0.35)	BB-

* Bonferroni Corrected p <0 .0017; E.S.: Effect Size; Chi-sq: Chi Square.

Southern Europe: Significant slight to moderate DIF (Class B) DIF was observed for G7. Motor Retardation, G12. Lack of Judgment and Insight, and G16. Active Social Avoidance. and G16. Active Social Avoidance favored the USA raters; G7. Motor Retardation, and G12. Lack of Judgment favored the Southern Europe raters. Items G2. Anxiety, G3. Guilt Feelings, G6. Depression, G10. Disorientation, G11. Poor Attention, G13. Disturbance of Volition, and G14. Poor Impulse Control, showed moderate to severe DIF (Class C) with G2. Anxiety, G3. Guilt Feelings, and G6. Depression favoring the USA raters, and G10. Disorientation, G11. Poor Attention, G13. Disturbance of Volition, and G14. Poor Impulse Control favoring the Southern Europe raters (see Table 6).

Russia & Ukraine: Significant slight to moderate DIF (Class B) DIF was observed for most items, G1. Somatic Concerns, G2. Anxiety, G3. Guilt Feelings, G6. Depression, G8. Uncooperative, G10. Disorientation, G13. Disturbance of Volition, and G14. Poor Impulse Control. G1. Somatic Concerns, G10. Disorientation, G13. Disturbance of Volition, and G14. Poor Impulse Control favored Russia & Ukraine, while G2. Anxiety, G3. Guilt Feelings, G6. Depression and G8. Uncooperative favored the US raters (see Table 6).

India: Table 6 shows, significant slight to moderate DIF (Class B) DIF was observed for G3. Guilt Feelings, and G14. Poor Impulse Control; G3. Guilt Feelings favored the US raters and G14. Poor Impulse Control favored the Indian raters. Significant moderate to severe DIF (Class C) were found for G2. Anxiety, G6. Depression, G10. Orientation, and G12. Lack of Judgment and Insight, with G2. Anxiety and G6. Depression favoring US raters and G10. Orientation, G12. Lack of Judgment and Insight favoring Indian raters.

Asia: Table 6 shows, significant slight to moderate DIF (Class B) for G1. Somatic Concerns, G2. Anxiety, G5. Mannerisms and Posturing; G9. Unusual Thought Content, G10. Disorientation, G11. Poor Attention, G12. Lack of Judgment/Insight, G13. Disturbance of Volition, and G14. Poor Impulse Control. G1, G9, G10, G11, G12, G13, and G14 favored Asian raters, while G5 and G16 favored US raters. Moderate to severe DIF (Class C) was observed for G6 Depression favoring US raters.

## Discussion

This article is the first to publish a cross-cultural comparison of the psychometric performance, mean scale scores, and item and scale-summary for the PANSS using qualified raters who rated one of 13 standardized training videos of a patient in the United States. Our aim was to perform a cross-cultural validity assessment by checking DIF due to cultural factor in a sample of qualified raters from 6 different geo-cultural groups. The results showed that there were significant differences in response to a number of items on the PANSS.

The Intra Class Correlations (ICCs) for the PANSS total score for the United States group was marginally higher than the ICC for the other geo-cultural groups. Although all reliability estimates were excellent (i.e., >= 0.80), all groups had the lowest ICCs for the Negative symptom subscale compared to the Positive symptom and General Psychopathology subscales suggesting increased variability among scores for the Negative symptom subscale.

### Rasch analysis and differential item functioning (DIF)

Although the PANSS was originally designed with three subscales (Positive, Negative, and General Psychopathology), studies examining the internal structure of the scale [59–61] have all identified the same two underlying factors, Positive and Negative. Other factors have varied and included Disorganized, Excitement, Hostility, Dysphoric, Catatonic and many more [15, 62, 63]. Given that Rasch analysis depend on how symptom severity is defined, the appropriateness of modeling of items via their subscale scores, rather than a total PANSS score was confirmed by conducting PCA on each subscale to assess unidimensionality. Although the PCA of the General Psychopathology subscale did not assume unidimensionality, it is common practice in clinical trials to examine the Positive and Negative subscales independently from the rest of the scale since these symptoms are considered a key component of the disease [15] and are symptom clusters which are primarily targeted in drug development.

While variation was present in the order and location of some PANSS items for geo-cultural groups, the overall pattern of item calibration was generally congruent. Within each of the six groups, the Rasch model also confirmed the hierarchical structure of the PANSS items, as evidenced by the pattern of average item calibrations and goodness-of-fit indices. In each region, most item calibrations were well spaced along the continuum of psychopathology, suggesting that for the groups included in this study, the PANSS is able to measure a wide range of function in schizophrenia. Items which were found to be easy to score by all geo-cultural groups included P3 (Hallucinatory Behavior), P6 (Suspiciousness/Persecutory Behavior), G12 (Lack of Judgment and Insight), and G2 (Anxiety). Additionally, results indicated that Northern European raters were more likely to endorse higher scores on all Positive symptom items except N7 (Stereotyped Thinking) compared to other regions. It should be noted, the first three items generally load on the Positive symptom factor domain in factor analytic studies [60, 61]. The Positive factor is comprised of the most active and first rank symptoms that define schizophrenia and it is primarily with these symptoms that a diagnosis is made clinically of schizophrenia. Therefore, raters may find it easier to score items which are first rank or core features of schizophrenia as these symptoms are also present in the diagnostic criteria.

In addition to the items listed above, raters from Northern Europe, the US and India found item N5 (Difficulty in Abstract Thinking) to be easier items to score. It should be noted that this item is intended to be based on objective responses by the patient, and not rater’s subjective interpretation. It can be suggested that items with clear scoring instructions related to objective response (e.g., if the subject answers four out of four proverbs correct, a score of one should be given) are easier to score across most geo-cultural groups. Other items which were observed to be easier to score include N2. Emotional Withdrawal (Southern Europe, and Asia), G12. Lack of Spontaneity and Flow of Conversation (Russia & Ukraine), G6. Depression (Northern Europe), and G16. Active Social Avoidance (United States of America, India, and Asia). With the exception of G6 (Depression), the latter items generally load on a Negative factor domain [60, 61]. The Negative factor reflects the difficulties in social relatedness often exhibited in many schizophrenic patients and are considered second rank symptoms. Again, more prevalent symptoms of schizophrenia and are first and second rank symptoms are easier to score across most countries. It should be noted that, upon evaluation of mean scores, Southern European raters scored higher on most Negative symptom subscale items (N1 Blunted Affect, N2 Emotional Withdrawal, N5 Difficulty in Abstract Thinking, and N6 Lack of Spontaneity and Flow of Conversation), whereas the lowest scores for Negative symptom items were from raters from Asia for N1 Blunted Affect, N3 Poor Rapport, N5 Difficulty in Abstract Thinking, and N7 Stereotyped Thinking compared to other geo-cultural groups.

All geo-cultural groups had significant DIF for P4 (Excitement), N7 (Stereotyped Thinking), and G10 (Disorientation). Santor and colleagues [28] have demonstrated that many items (N7 and G10) have problematic features and some fundamental issues with relation to the level of psychopathology measured by the overall PANSS. Our own item response analysis [30], has also demonstrated significant DIF with item G10 (Disorientation) with regards to its contribution to the assessment of psychopathology as measured by the PANSS. Additionally, previous psychometric investigations have indicated that item G10 (Disorientation) either does not discriminate well in terms of assessing overall severity or does not reflect dimensional individual differences between patients within schizophrenia [15, 62]. Similarly, all groups showed significant slight to moderate DIF with the US raters for item G10 (Disorientation). This item measures the lack of awareness of the subject’s relationship to their surroundings and assesses specific questions relating to the subject’s knowledge of his/her doctor, address, and political figures. Therefore, this item may also lack a relationship to psychopathology, rather than cultural/geographical differences in scoring patterns.

The main source of rater differences among items were observed for the General Psychopathology subscale with five items showing different rating patterns across geo-cultural groups (i.e., G3 Guilt Feelings (Russia & Ukraine), G5 Mannerisms and Posturing (Asia), G16 Active Social Avoidance (Southern Europe and Russia & Ukraine), G14 Poor Impulse Control (all regions except Northern Europe), G15 Preoccupation (Russia & Ukraine)). Although support for the PANSS General Psychopathology subscale has been found in other studies [13], the current findings suggest the rating for items on the General Psychopathology subscale differ for European and Japanese raters and it should not be assumed that the same, standard rating tools were applied indiscriminately across these groups.

There are several possible explanations for discrepancies among raters both between and within the geo-cultural groups examined [63]. One of the possible reasons may be interpretation variance. Interpretation variance implies that once raters agree on the common criteria, when there are differences, it is more frequently because of decision-making differences in the scoring of the item. Thus, when training a cohort of raters, it may be necessary to focus part of the training on cultural differences and expectations on different thresholds of symptoms. There were significant moderate to large DIF (i.e., ETS Class scores of C) for most items scored by Southern European raters compared to US raters. Different social views due to cultural influence might lead to different rating of social and emotional behaviors. Despite the fact that the Positive and Negative subscales met the other Rasch model requirements, the presence of DIF by region means that culture might contribute to the scores on these items. Therefore, when clinical trial investigators pool PANSS data from different countries, items showing DIF should be removed or split. An iterative “top-down purification” splitting approach for items showing uniform DIF has been applied elsewhere [26].

Yet another possible source of differing reliability may be cultural biases found in common-place standardized training methods and materials. Using standardized patients (patients who are trained to portray specific sets of symptoms), it has been demonstrated that video-recorded or tape-recorded interviews increases inter-rater reliability even among raters with limited exposure to the PANSS (e.g., [64]) and affective measures [65]. As such, a culturally diverse group of raters was asked to evaluate cultural idioms, symptom expressions, and social dynamics during the interview which may have been unfamiliar. It can be argued that higher inter-rater reliability may therefore be associated with a higher degree of acculturation amongst raters as much as it is an indication of rater comprehension and agreement.

### Limitations

There are some limitations to this study. First, this analysis focuses on a small cohort of raters from only six geo-cultural groups who rated US training videos. This sample is not representative of all PANSS raters and patients within the geo-cultural groups (except the US); hence findings may not be generalizable across different regions (e.g., South America, Latin America, and areas of Africa). Also, a similar analytic technique using data obtained in clinical trials (utilizing patients from the specific geo-cultural region) is currently underway by this research team. Secondly, this study addresses reliability cross-sectionally, and not longitudinally. Additional studies would be needed to address differences in PANSS scores across time. Unfortunately in this analysis, we were unable to access data to adjust for possible confounding variables such as level of rater qualifications, amount of experience in the field of schizophrenia, or gender, and recognize that these may influence the differences in mean scale scores. This is, however, a common limitation of cross-cultural comparisons of any subjective or objective data where local socio-demographic conditions vary in their definition and measurement. Since this is the first cross-cultural study of the PANSS and not taking into account the presence of confounding sample characteristics other than geographically defined culture, these findings should to be taken in a preliminary and cautionary manner. More specifically, how rater training is translated in the geo-cultural regions could not be examined using the currently available data, and should be addressed in future cross-cultural studies. Additionally, culture may influence responses to the PANSS for many reasons. For example, some Hispanics have been noted to express emotional and mental health as physical health symptoms [66], which may be different from non-Hispanics. Also, Klienman and Good [67] reported individuals with depression may be less likely to report sadness or anxiety, but more likely to report sleep problems or appetite changes. The authors recognize that wide variations exist in educational level, occupational status, and cultural identity within communities of raters, therefore as much as geo-cultural matching was attempted, the authors recognize the limitations of the selection of countries per group. Finally, although Rasch analysis allows for the detection of DIF within the current sample size, future studies should attempt to replicate these results using greater and more balanced sample sizes across regions.

## Conclusions

This is the first Rasch analysis of the PANSS in a global setting across cultures. One strength of the Rasch analysis is that problematic items are clearly flagged and specific modifications can be identified to improve rater training and data surveillance of the PANSS. The results showed support for the two subscales (i.e., Positive and Negative symptoms) with recommendations to further assess administration and scoring of specific items; however, the General Psychopathology subscale was shown to be a multidimensional subscale warranting further review. Attention to cultural bias in training curricula and delivery may help to reduce these elements as confounders in future inquiries. The results of the current study further emphasize the need for rigorous individualized training and rater surveillance of scores on the PANSS across different groups, to decrease sources of unreliability. Clearly further research is warranted to confirm these findings and establish good sensitivity and specificity across cultures.

## Authors’ information

AK is a Statistician at ProPhase LLC and Manhattan Psychiatric Center, New York, NY; she has 10 years’ experience working in psychopharmacology research as a statistician and has peer reviewed publications in clinical trials in schizophrenia, including collaborations on book chapters. AKs research interests are in Item Response Theory, Testing and Measurement and Bayesian applications in clinical research. AK obtained a degree in Psychometrics from Fordham University, NY. CY studied at Manchester Metropolitan University, UK, and is the current Clinical Director at CROnos CCS. CYs research interests are testing and measurement, data monitoring, surveillance and mental health studies. CY has publications in statistics, data monitoring and testing and measurement. MO is the Chief Executive Officer at ProPhase LLC and holds an academic position at New York University, NY. SL has a PhD in Middle Eastern and Islamic Studies and is a former Fulbright Scholar and the Lead Research Consultant at The PANSS Research Institute, Inc. GDC is the Chief Executive Officer of CROnos Clinical Consulting Services. His interests are in data monitoring. He also contributes to rater training efforts for clinical trials. BR is a Research & Training Associate at ProPhase LLC; he obtained his degree in Clinical Psychology at Long Island University, New York, NY and is a licensed Psychologist in New York. His interests include testing rater training and clinical psychology. LL is a Research & Training Associate at ProPhase LLC and an Assistant Professor at Pratt Institute, Brooklyn, NY. His research focuses on cross-cultural comparisons, language and development. SJ is the President of ProPhase LLC and obtained her doctorate degree in Clinical Psychology from Long Island University, New York. Her interests are in rater training and clinical psychology. TI is a medical doctor and the Vice President of Seiwa Hospital, Institute of Neuropsychiatry in Tokyo, Japan. His work includes adenosine A2A receptor associated methamphetamine dependence in Japanese, cross cultural comparisons, and pharmacological treatments for Japanese patients with schizophrenia. LY is an epidemiologist at Columbia University, Mailman School of Public Health, New York. LYs work focuses on several key areas of psychiatric epidemiology including identification of subtypes of schizophrenia, cultural implications of schizophrenia, developing interventions for Asian Americans with psychosis.
